# Developmental Coordination Disorder: State of the Art and Future Directions from a Neurophysiological Perspective

**DOI:** 10.3390/children9070945

**Published:** 2022-06-24

**Authors:** Marco Emanuele, Giovanni Polletta, Maddalena Marini, Luciano Fadiga

**Affiliations:** 1 Section of Physiology, Department of Neuroscience and Rehabilitation, University of Ferrara, via Luigi Borsari 46, 44121 Ferrara, Italy; mnlmrc@unife.it (M.E.); g.polletta@libero.it (G.P.); 2 IIT@UniFe Center for Translational Neurophysiology of Speech and Communication (CTNSC), Istituto Italiano di Tecnologia, via Fossato di Mortara 19, 44121 Ferrara, Italy; maddalena.marini@iit.it

**Keywords:** developmental coordination disorder (DCD), neurodevelopmental disorders, transcranial magnetic stimulation (TMS), motor development, motor control

## Abstract

Developmental coordination disorder (DCD) is a common neurodevelopmental condition characterized by disabling motor impairments being visible from the first years of life. Over recent decades, research in this field has gained important results, showing alterations in several processes involved in the regulation of motor behavior (e.g., planning and monitoring of actions, motor learning, action imitation). However, these studies mostly pursued a behavioral approach, leaving relevant questions open concerning the neural correlates of this condition. In this narrative review, we first survey the literature on motor control and sensorimotor impairments in DCD. Then, we illustrate the contributions to the field that may be achieved using transcranial magnetic stimulation (TMS) of the motor cortex. While still rarely employed in DCD research, this approach offers several opportunities, ranging from the clarification of low-level cortical electrophysiology to the assessment of the motor commands transmitted throughout the corticospinal system. We propose that TMS may help to investigate the neural correlates of motor impairments reported in behavioral studies, thus guiding DCD research toward a brain-oriented acknowledgment of this condition. This effort would help translational research to provide novel diagnostic and therapeutic tools.

## 1. Introduction

Developmental coordination disorder (DCD) is a condition that includes delays in the acquisition of motor skills, clumsiness, slowness, and inaccurate execution of everyday life tasks (e.g., catching objects, using scissors, handwriting, riding a bike) [1,2]. These difficulties are not secondary to intellectual disability or medical conditions, such as cerebral palsy or muscular dystrophy. Symptoms appear during early development and spread beyond infancy, negatively impacting daily activities, such as dressing and feeding, school performance and social interactions [3,4].

DCD is considered a continuum of disorders, rather than a discrete category. Individuals with DCD are comprised in the low tail of the normal distribution of motor skills [5]. The prevalence of this condition is strictly associated with the criteria used in each study, varying from 1.4% to 19.0% of school-age children [6,7,8], while the ratio of males to females ranges from 2:1 to 7:1 [9]. Alongside with male sex, preterm birth represents the major risk factor for DCD [10,11].

Motor control impairments in DCD received substantial attention over the last decades. The emerging picture displays a broad range of abnormalities, spanning from how voluntary actions are planned, to their control and monitoring during motor execution [5,9,12]. Additional problems involve motor learning and imitation [13,14,15]. Despite these remarkable achievements, DCD research still lacks neurophysiological data. Functional magnetic resonance imaging (fMRI) studies indicate that a widespread neural network might be involved in this condition [16]. However, the intrinsic limitations of this technique (e.g., low temporal resolution, poor versatility) prevent a complete understanding of the neural correlates of DCD.

This review pursues a double scope. Our first aim is to provide a survey of previous literature addressing motor impairments in children and adults with DCD. We chose not to discuss these impairments through the lens of a specific model of motor control (e.g., internal models, motor synergies, etc.). Rather, we aim to provide readers with a comprehensive overview of the general processes involved in motor control (planning and monitoring of actions, motor learning, action observation and imitation) and their alterations in DCD. This was done in the attempt to reach a readership of both researchers and clinicians accustomed to deal with DCD in everyday practice but possibly not familiar with theoretical models of motor control. References to specific models of motor control are made when useful to discuss particular motor problems, such as motor planning and motor imagery impairments (often interpreted as impaired internal modelling of actions) and degrees of freedom control (which will be discussed using the motor synergies framework).

Noteworthy, many aspects of motor control that are addressed in DCD research by means of behavioral measures were successfully investigated in healthy subjects using transcranial magnetic stimulation (TMS), since it was introduced in 1985 [17]. Therefore, our second goal in this review is to illustrate the potential applications of this technique in DCD research. TMS is a non-invasive brain stimulation technique based on the principle of electromagnetic induction. A brief current pulse passing through a coil held over the scalp causes a magnetic field that penetrates the skull and excites the nervous tissue [18,19]. TMS allows ongoing cortical activity to be sampled during cognitive and behavioral tasks, thus providing a method to investigate intracortical circuits, including their excitatory and inhibitory components. This technique may help to unveil the neurophysiological mechanisms that contribute to abnormal motor behavior in DCD. In contrast with other neurodevelopmental disorders (e.g., autism and attention deficit with hyperactivity disorder [20,21]), TMS is yet rarely use in DCD research. Bridging this methodological gap may help to expand and refine our understanding of DCD and to implement novel diagnostic approaches and interventions.

## 2. Planning and Monitoring of Actions

Voluntary movements are seamlessly generated, monitored, and re-corrected by the brain according to behavioral goals and (time-varying) contextual cues. Alterations in these processes are believed to cause motor control impairments in DCD.

To begin a movement, behavioral goals need first to be converted into motor commands. This process is commonly known as motor planning [22]. To achieve this, the brain must represent the desired/expected outcome of a movement along with the current state of the effector (e.g., the upper limb for reaching-grasping movements). This allows the most appropriate set of motor parameters to be selected among a vast number of different alternatives, thus transforming the current state of the effector into the desired outcome. For example, one may have to choose whether to grasp an object with the right or left hand or using a power grip vs. a precision grip. Importantly, this decisional process relies on predictions about the future course of voluntary actions. That is, deciding how to grasp an object (e.g., with the right or left hand) is influenced by the manipulation or overall purpose one wants to gain after having picked up the object [23,24].

The selection of motor parameters is further complicated by the redundance of degrees of freedom in the motor system, allowing multiple goal-equivalent solutions for most everyday life motor tasks [25]. For example, it is possible to perform a pointing movement through multiple goal-equivalent trajectories. In addition, the same trajectory can be obtained through many different sequences of muscle activation and joint rotation. To solve the problem of redundancy, degrees of freedom are grouped into motor synergies and controlled together rather than individually, thus reducing the number of variables involved in motor control [26]. Each motor synergy can be scaled and combined with other synergies, producing widely differentiated and flexible motor behaviors [27].

Once a movement is initiated, the motor plan can be updated by making online corrections to counteract unexpected perturbations [28]. For example, consider the situation in which one wants to grasp an object poised at the edge of a table. If the object starts falling towards the ground after they started reaching for it, the motor plan must be updated ‘on the fly’ to catch the object before it hits the floor. As voluntary movements are often performed in a dynamic environment that may change unexpectedly (like in the previous example), flexible shifts from one motor plan to another in the presence of sudden perturbations are crucial for efficient motor behavior.

In parallel to overt movement execution, motor behavior entails an intricate pattern of covert neural activity that helps to monitor unfolding movements and to anticipate their outcome and consequences. Specifically, during movements, a copy of the motor commands dispatched to the muscles (i.e., the efference copy) is relayed throughout many different neural substrates [29,30]. The efference copy contributes to the creation of internal representation of actions (or internal models) that support the generation of effective motor commands [31].

Finally, accurate movements require that the brain handles noise and variability in neural circuits. Variability is an inherent, unavoidable characteristic of motor behavior. For example, a pointing movement will never hit the target exactly in the same position when repeated over and over. These trial-to-trial fluctuations of motor performance likely origin (at least in part) from random noise in neural signals [32]. As with every signal, neural signals are indeed affected by noise, i.e., unwanted random information that adds to the information sent through neural circuits, interfering with it just as white noise would affect the sound of a radio. Excessive noise may lead to jerky and unpredictable motor performance, possibly impacting the acquisition and refinement of motor skills during early development.

In this section, we will survey studies that investigated the processes involved in the planning and monitoring of goal-directed actions in DCD from different perspectives, including the selection of motor parameters, the coordination of redundant degrees of freedom, online corrections in response to unexpected perturbations, internal modelling of actions (as indexed by motor imagery) and motor variability.

### 2.1. Predictive Selection of Motor Parameters

Many everyday life tasks involve reaching-grasping objects or tools and manipulating them to achieve specific behavioral goals (e.g., picking up an upside-down glass to pour some water). Normally, the selection of the grip strategy (i.e., the configuration/orientation of the hand while grasping the target object) aims for a comfortable position of the upper limb while manipulating the object, even if it comes at the cost of adopting an awkward limb posture during grasping [23,24]. This effect is known as end-state comfort and requires using predictive information about the final goal of the action to select the most appropriate grip strategy (i.e., anticipatory planning). For example, grasping an upside-down glass to pour some water would be most likely done with a thumb-down rather than a thumb-up hand orientation. This facilitates a comfortable posture (and control) of the upper limb while pouring the water.

Children and adults with DCD often fail to use end-state comfort as a grip selection strategy, suggesting poor anticipatory planning skills [33,34,35,36,37]. On the contrary, they tend to minimize the initial rotation of the wrist, possibly leading to an awkward final hand posture that likely compromises control. For example, Adams and colleagues employed a reaching-grasping task in which participants were instructed to pick up a wooden sword presented in different orientations and stick it into a horizontal slot [34,37]. Typically developing (TD) children planned the initial grip of this motor sequence aiming to a comfortable final posture of the hand, even when this strategy required a complex initial rotation of the wrist while grasping the sword. Conversely, children with DCD minimized the initial rotation of the wrist, leading to an awkward final posture of the hand.

Predictive information is also used in motor planning to generate anticipatory postural adjustments, defined as the electromyographic activity recorded from postural muscles before the onset of a movement [38,39]. The functional meaning of this activity is to anticipate postural perturbations secondary to limb motion and prevent any loss of balance. Children with DCD showed impaired anticipatory postural adjustments in several motor tasks, including pointing movements [40], grasp-and-lift sequences [41] and voluntary unloading tasks in which participants used the dominant hand to remove a load that was attached to the non-dominant hand [42]. For example, in pointing movements, the activation of postural muscles that stabilize the trunk resulted delayed in children with DCD compared to TD children [40].

In sum, altered grip selection and impaired anticipatory postural adjustments suggest that anticipatory planning is impaired in DCD. Noteworthy, while grip selection is related to the control of a distal effector (the hand), anticipatory postural adjustments involve proximal and postural control. Anticipatory planning impairments seem thus pervasive in DCD, broadly affecting different domains of motor control.

### 2.2. Motor Synergies and Degrees of Freedom Control

Studies on catching movements demonstrated distinct strategies of degrees of freedom control in DCD [43]. For example, children with DCD showed stronger coupling between different upper limb segments (i.e., the hand, the wrist, the elbow, and the shoulder, both within and between limbs) compared to TD children when they caught a ball with both hands [44]. Authors suggested that TD children are able to explore multiple motor solutions among those available rather than imposing a rigid coupling on degrees of freedom as DCD children. Accordingly, in TD children, degrees of freedom are handled in a more adaptable and flexible manner as opposed to children with DCD. In a similar task, children with DCD displayed smaller and less variable excursion of the elbow joint than TD children [45]. In this way, most of the degrees of freedom involved in the movement were blocked, leaving just one or few more controlling the displacement of the end-effector. In other words, children with DCD “froze” the elbow, rather than coupling the shoulder and the elbow in a flexible motor synergy. Importantly, although this strategy simplified motor control by reducing the number of degrees of freedom involved in the movement, it also led to lower accuracy in the task (i.e., less balls effectively caught). In a similar vein, studies on prehension movements reported a temporal decoupling of reaching and grasping in children with DCD [46,47]. In TD children, the maximum grip aperture was consistently time-locked to the peak of deceleration of the wrist; in contrast, in children with DCD the maximum grip aperture occurred earlier during reaching and with more variable timing [46]. Again, this control strategy allows children with DCD to reduce the number of degrees of freedom simultaneously active during movements, suggesting poor ability to combine and coordinate multiple degrees of freedom.

The consequences of impaired motor synergies and degrees of freedom control can be far-reaching. During motor development, existing motor synergies can be rescaled and combined together to map new sequences of motor commands [48]. This helps children to improve effortlessly their motor repertoire and acquire new motor abilities. Consequently, impaired synergies may impact this process, leading to delayed motor development. In addition, as we will discuss later, the impairment of motor synergies may explain (at least in part) abnormal motor variability in DCD.

In sum, degrees of freedom control seems impaired in DCD, leading to distinct strategies for reducing the number of degrees of freedom simultaneously active during movements. Motor synergies are a cornerstone of motor control, which allows to handle redundant degrees of freedom and find unambiguous solutions for motor tasks. Therefore, this impairment may have important implications in motor behavior that should be further addressed in future studies.

### 2.3. Online Corrections

Online corrections in DCD were mostly investigated using the double-step paradigm, in which subjects aim (e.g., through a pointing or a sliding movement) for a target projected on a screen that can unexpectedly shift its position [49,50]. This requires adjusting the pre-planned movement trajectory ‘on the fly’. In versions of the double-step paradigm involving pointing movements in 3-dimensional space, children with DCD performed poorly compared to TD children, with slower movement time and, most importantly, delayed onset of corrective movements in trials in which the target shifted its position [51,52,53]. These online control difficulties were exacerbated in a modified version of the task, in which corrections had to be performed towards an opposite direction with respect to the target shift [54]. This manipulation added an inhibitory load to the double-step paradigm because children were required to hold automatic re-programming towards the target and generate a movement in the opposite direction. Difficulties in shifting from one motor program to another may thus originate from problems in inhibitory control, leading to delayed suppression of the ongoing motor plan. Alternatively, impairments of online corrections may reflect more general problems in mapping behavioral goals onto motor commands. Effective corrections crucially require fast re-computing of the new target into motor coordinates. Therefore, (previously discussed) difficulties in computing in advance the appropriate motor parameters for a given behavioral goal and in coordinating motor degrees of freedom may affect the performance in the double-step paradigm.

Results supporting impaired online corrections were not replicated using a 2-dimensional version of the double-step paradigm, in which participants aimed for the target through a sliding movement performed on the surface of a touch screen [34]. In this version of the task, children with DCD produced online corrections as effectively as TD children. Two (non-mutually exclusive) explanations were proposed: (1) lower task demands, as the sliding movement was performed in a 2-dimensional space, whereas the pointing tasks required 3-dimensional movements; (2) the heterogeneous severity of motor impairments among children recruited for different studies.

In sum, children with DCD performed worse than their TD counterparts in most of the studies that assessed online corrections, except for one study that employed a version of the double-step paradigm involving 2-dimensional movements. This indicates poor flexibility in motor control, which is likely to affect the execution of everyday life movements and contribute to the overall deficits in motor performance.

### 2.4. Efference Copy and Motor Imagery

Motor imagery constitutes a unique case in which internal representations of actions are produced without any action being actually executed. Therefore, motor imagery provides a privileged window to investigate the efference copy and internal representation of actions.

In children with DCD, motor imagery was widely investigated using the hand rotation paradigm, in which participants are asked to judge as fast and accurately as possible the laterality of a hand projected on a screen in various orientations [55,56]. Normally, reaction times are influenced by the physical constraints of the actual movement that would be required to match the observer’s own hand to the one displayed on the screen. For example, reaction times are generally slower when the hand on the screen is rotated outwards (i.e., laterally, away from the body midline) than inwards (i.e., medially, toward the body midline), likely because lateral hand rotations are generally more difficult for biomechanical reasons [55,56,57]. Changes in reaction times according to hand orientation led researchers to speculate that the same brain resources that would be needed to match the observer’s hand with the one on the screen are exploited to solve the task (being thus affected by similar constraints). In children with DCD, reaction times are slower and less affected by the orientation of the hand presented on the screen, suggesting poor motor imagery skills and impaired generation of the efference copy [58,59,60,61,62]. Motor imagery difficulties in children with DCD were proportional to the task demands in terms of complexity of the imagined movement. For example, children with DCD performed worse in tasks that required simulating a hand rotation on two axes rather than on only one axis [60]. In addition, their performance was influenced by the severity of the clinical outcome: Children with mild DCD experienced some benefits in terms of task accuracy from the explicit instruction of using motor imagery to accomplish the task; no such advantage was observed in children with severe DCD [61,62]. Interestingly, notwithstanding being less capable than TD children of using motor imagery to solve the hand rotation paradigm (as indexed by reaction times being poorly affected by hand orientation), in some studies children with DCD performed as accurately as their TD peers in judging hand laterality [59]. It was proposed that children with DCD may rely (at least partially) on alternative strategies to solve this task. For example, they may judge handedness based on perceptual cues, rather than mapping the task onto motor coordinates.

Another paradigm that was used to assess motor imagery in DCD is called the visually-guided pointing task. This task was originally developed to investigate motor imagery in patients with parietal lesions [63]. Participants either move or imagine moving the tip of a pen back and forth a target area of variable size. Both in children with DCD and TD children, the duration of real movements was affected by the target size as predicted by Fitts’ law [64,65,66,67]. This well-established motor control law states that movement time is proportional to the logarithm of the ratio between movement length and target width [67]. The target size also influenced the duration of imagined movements in TD children, but not in children with DCD [64,65,66].

Taken together, these results indicate that in DCD motor imagery is not affected by the same constraints that influence actual action execution. It follows that the efference copy mechanism and, more generally, internal representation of actions, seem not very useful to convey information for motor control purposes. The impairment of the efference copy mechanism—along with findings suggesting poor anticipatory planning and altered online monitoring of movements—led to the formulation of the so-called internal modelling deficit hypothesis [68]. According to this hypothesis, motor impairments in DCD arise from impaired internal simulation of actions through the so-called internal models. The theoretical ground for the internal modelling deficit hypothesis relies on a computational formalization of the motor system provided by Wolpert and colleagues [31]. In brief, different types of internal models (i.e., brain representations of actions) are used to describe motor control processes, including planning and monitoring of actions. According to the internal modelling deficit hypothesis, motor deficits in DCD arise from impaired encoding of internal models, leading to the selection of abnormal motor parameters for motor behavior.

### 2.5. Noise and Variability

On top of the motor control impairments that we have described so far, motor symptoms in DCD are likely influenced by abnormal noise affecting the brain circuits involved in motor control [69]. Findings suggesting augmented variability in motor performance were observed in many different tasks. More specifically, in children with DCD, aiming movements showed jerkier and more variable paths as opposed to TD children [70,71]; during steady, isometric muscle contraction, the force output was unstable and poorly consistent across trials [72]; gait patterns measured at the shank and thigh level were more variable from stride to stride [73] and, similarly, inter-clap and inter-footfall intervals were more variable when clapping movements were performed while marching in place [74].

Increased motor variability in DCD may originate from altered strategies for degrees of freedom control, such as individual control of degrees of freedom (e.g., obtained by reducing overlapping activation of multiple degrees of freedom) as opposed to synergistic control. Mapping actions directly onto redundant degrees of freedom (instead of grouping degrees of freedom into motor synergies) results in ambiguous solutions, leading to scattered performance when actions are repeated over and over. Alternatively, abnormal levels of neuromotor noise may derive from impaired inhibitory control in brain circuits [75]. As we discussed above, alterations in inhibitory control were suggested to cause difficulties in online corrections. Further studies are needed to investigate whether a similar inhibitory deficit may also be responsible for augmented motor variability in DCD.

Increased neuromotor noise and abnormal variability entail uncertainty and ambiguity in motor behavior. In turn, this may impact the formation of robust sensorimotor representations. Motor control develops and refines during the early interactions with the external world, leading to complex and widely differentiated sensorimotor representations [76,77]. Voluntary actions are initially gross and poorly differentiated. However, repetitive interactions with the external world affordance help infants to gradually tailor motor parameters to accomplish increasingly specific and demanding tasks. Abnormal variability may disrupt the link between motor commands and their outcomes, leading to scattered and broadly tuned sensorimotor maps [69,78].

## 3. Motor Learning by Trial and Error

Among many defining features of DCD [1], a major one is constituted by delayed learning of motor skills that are expected at a given chronological age. Some studies suggest that motor delays may (at least in part) depend on alterations affecting motor learning by trial and error [70,71,79,80]. Over subsequent attempts, this learning mechanism gradually sculpts the motor commands that orchestrate a motor task based on the discrepancy between its expected and actual outcome [31,81,82,83]. For example, in the so-called prism adaptation task, individuals engaged in darts throwing show gradual adaptation to a distortion of the visual feedback obtained through prismatic spectacles that shift the visual field of a certain angle [84]. After some trials in which they miss the target of an angle corresponding to the visual feedback distortion, participants normally manage to execute accurate throws and eventually hit the target. When the visual distortion is removed, subjects miss the target again, this time of an opposite angle with respect to the previous visual distortion (i.e., visual distortion aftereffect). The aftereffect indicates that a new visuomotor map was established during the exposure to the distorted visual feedback, thus providing a measure of motor learning.

The results of studies that investigated visuomotor adaptation to visual feedback distortion in DCD suggest impaired learning by trial-and-error in this condition. In a study in which participants received visual feedback while drawing on a digitized tablet, children with DCD showed slower adaptation to a 45° feedback distortion and no aftereffect when the distortion was removed [70]. In a follow-up experiment, a stronger 60° distortion was able to produce an aftereffect in children with DCD, yet this was smaller compared to TD children [71]. Two other studies that employed the prism adaptation task retrieved mixed results. In one study, children with DCD showed a slower rate of adaptation to visual feedback distortion, though with a similar aftereffect as TD children [79]. In another study, no group effect was observed in the rate of adaptation, although the larger performance variability that was detected in the DCD group could have hidden adaptation problems [80]. Nonetheless, individual subject analysis showed slower adaptation to visual feedback distortion or absent aftereffect in most of the participants in the DCD group.

In conclusion, children with DCD show impairments of motor learning, preventing them from tuning their actions based on previous errors. These problems may depend on a specific impairment of the brain mechanisms serving this type of motor learning; in this direction are fMRI studies showing impaired activation of cortical-cerebellar circuitry [85,86,87,88], a well-established neural substrate for motor learning by trial-and-error [83,89]. Alternatively, motor learning impairments may originate from the alteration of action representation in the brain. Updating of motor commands based on error information must crucially rely on solid internal representation of actions, including accurate predictions about their outcome. If internal models of actions are weak or imprecise (as proposed in the internal modelling deficit hypothesis), this learning process cannot take place.

## 4. Praxis and Imitation: Mirror Neurons Involvement in DCD

Studies that assessed praxis in children with DCD (mostly by means of behavioral scoring systems derived from neuropsychological research) showed difficulties in the imitation of everyday life gestures, such as teeth brushing, hair combing, waving goodbye or blowing a kiss [90,91,92,93,94]. These results suggest problems in mapping observed actions onto their own motor repertoire.

One possible explanation for imitation problems in DCD could be an impairment of the so-called mirror neuron system [14,15]. Mirror neurons are a subset of visuomotor neurons originally discovered in the area F5 of the monkey ventral premotor cortex [95,96]. The firing rate of mirror neurons increases both when the monkey performs an action and when it observes another individual (human or non-human primate) executing a similar action. Neural networks with similar discharge properties were also detected in humans using neuroimaging techniques [97,98], electroencephalography [99,100,101,102,103,104], magnetoencephalography [105] and TMS [106]. On the other hand, imitation problems in DCD may also originate from the distinct organization of self-initiated actions. The peculiar motor repertoire of children with DCD may reduce the kinematic similarity with other individuals. In turn, this may impact the ability of representing and understanding others’ actions [107].

Praxis studies provided indirect (behavioral) evidence of abnormal representation of others’ actions in DCD. In contrast, a recent fMRI study addressed the neural underpinnings of these findings, showing decreased activation of the cortical regions associated to the human mirror neuron system (i.e., precentral gyrus, inferior frontal gyrus, posterior cingulate and precuneus complex) in children with DCD compared to TD peers during the observation of a finger sequencing task [108]. However, these results were not replicated in a follow-up study by the same group [109], highlighting the need for further research in this field.

Impaired representation of others’ actions may have important implications. TD children are capable of complex and flexible representation of others’ actions, which allow them to explore novel motor strategies and enrich their motor repertoire [110]. In contrast, children with DCD may have difficulties in extracting motor information from others’ movements that may be useful to improve their own. Alongside problems in motor learning by trial-and-error, this may contribute to delays in the acquisition of motor skills. In addition, problems in representing others’ actions may reduce the ability to cooperate in socio-motor behaviors (e.g., in joint tasks) [111,112].

In conclusion, difficulties in action imitation in DCD indicate problems in mapping actions observed in other individuals, which may have important implications for motor learning and socio-motor interaction. These problems may arise from a dysfunction of the mirror neuron system or, more broadly, from a distinct organization of the motor repertoire.

## 5. Neural Correlates of DCD: From Early Hypotheses to Brain Imaging

As we have discussed so far, research in DCD has gained important results over recent years. However, some issues remain open, mainly concerned with the neural mechanisms of motor control impairments. Which functional abnormalities in neural circuits contribute to motor control difficulties in DCD? Do the motor commands transmitted by the motor cortex carry any specific alteration? Are the excitatory/inhibitory circuits of the motor cortex involved in causing these alterations? These—among others—questions were not yet fully clarified. In this section, we will briefly survey the early hypotheses about the brain basis of DCD and the major fMRI findings in this condition. In the next section, we will illustrate the potential applications of TMS in this field. This technique may help to clarify the motor problems reported in behavioral studies, overcoming at the same time some of the limitations imposed by brain imaging.

Initial speculations on the neural correlates of DCD were based on the observation of shared characteristics between children with DCD and patients carrying brain lesions in the cerebellum, basal ganglia, and parieto-frontal cortex [113,114]. For example, children with DCD perform worse than TD children in routine tests of cerebellar function, such as finger-to-nose touching and rapid alternating hand movements [113,115]. Likewise, findings such as higher variability in rhythmic movements [116] and impaired motor learning by trial-end-error [70,71] are remarkably similar to those seen after cerebellar lesions [117,118]. Some other motor problems in DCD resemble those observed in patients with dysfunctions of the basal ganglia (e.g., Parkinson’s disease). For example, children with DCD are less able than TD children to produce isometric force pulses of consistent amplitude from trial to trial [113]. At the same time, problems in motor imagery, action planning and motor sequences were linked to alterations in fronto-parietal circuits [68,114].

Following these speculative attempts to link motor problems in DCD with some specific brain circuits, more solid evidence was gathered through brain imaging techniques. Results of fMRI studies in DCD were extensively reviewed elsewhere [16]. In brief, children with DCD show abnormal brain activation in both cortical (e.g., the fronto-parietal cortex) and subcortical structures (i.e., the basal ganglia and the cerebellum) [85,86,87,88,119,120,121]. These results indicate that DCD does not depend on the impairment of a specific brain region, but rather from distributed alterations in neural activity. Considering this, fMRI carries the important advantage of providing functional information from the whole brain, including the activation of widely distributed brain circuits in specific behavioral contexts. However, these studies have important limitations, too. First, fMRI cannot capture the temporal dynamics of neural networks. Second, brain imaging is not informative about the fine properties of intracortical circuits, such as the balance between inhibitory and facilitatory synaptic inputs. Third, these studies require complex and time-consuming data analysis, which might be unfeasible for translational applications.

In conclusion, brain imaging has allowed researchers to gather important knowledge on the neural basis of DCD, mostly confirming early speculations based on the similarity between motor findings in DCD and those seen in patients with neurological lesions. Yet, due to the technical limitations of brain imaging, this approach may prevent a full disclosure of how altered brain activity causes the motor symptoms found in DCD.

## 6. Future Directions: Transcranial Magnetic Stimulation

Brain imaging limitations can be overcome by combining the results gathered using this approach with non-invasive brain stimulation techniques, such as TMS. This technique allows us to investigate specific components of cortical circuitry and provides temporally accurate information on the dynamics of brain activation. Importantly, no major side effects (e.g., seizures) were observed using TMS in newborns, children, and adolescents, while mild and transitory side effects (e.g., headaches, twitching, fatigue, mood changes, scalp discomfort) appeared in a few participants [122,123,124]. In addition, auditory side effects related to the loud ‘click’ noise produced by TMS pulses were carefully ruled out [125]. Despite being a safe and convenient technique, TMS is still rarely used in DCD research. Yet a broader application of this technique in DCD research may help to develop more accurate models of this condition (i.e., including its neurophysiological basis), and possibly support translational applications.

Since its introduction [17], several TMS protocols have been developed in clinical and research settings [18]. When applied to the motor cortex, TMS elicits electromyographic responses known as motor evoked potentials (MEPs) [18,126,127]. MEPs are observed some 20–25 ms after the magnetic stimulus in those muscles that have their cortical somatotopic representation within the stimulated area. Their amplitude provides a measure of corticospinal excitability, which is influenced by the balance between excitatory and inhibitory inputs to the motor cortex at the time of stimulation [126,127,128]. By exploiting these quite simple electrophysiological responses, over the last three decades TMS has helped to clarify several neural mechanisms that shape motor behavior in humans.

For example, MEPs provide an accurate readout of the cortical dynamics occurring during motor planning [129]. Many everyday life activities require us to choose among different available motor solutions to achieve behavioral goals. We have previously discussed that this process is impaired in children with DCD, as demonstrated by impaired modelling of actions based on predictive information (e.g., they do not use end-state comfort as a constraint for motor planning and show impaired anticipatory postural adjustments). In studies in which healthy adult participants chose between two possible motor responses, MEPs recorded during the reaction time following a cue signal were facilitated in muscles selected for the upcoming response and suppressed in unselected muscles [130,131]. A similar approach may help to decipher the neural correlates of action selection and motor planning in DCD.

Individuals with DCD also show impaired online corrections, possibly depending on impaired inhibitory control [54]. In addition, reduced inhibition may facilitate neuromotor noise. In brain circuits, inhibitory signals support focal processing of information and precise timing of neural activation [75]. Reduced inhibition may result in more variable and scattered activation of neural networks, leading to higher motor variability. TMS provides a useful tool for assessing intracortical inhibitory circuits. MEPs elicited during concurrent muscular activation are followed by a period of electromyographic suppression know as cortical silent period (cSP) [132]. The cSP is mediated by inhibitory components of intracortical circuitry, mainly acting on GABA_B_ receptors [133]. Accordingly, it provides a valuable index to investigate intracortical inhibition during ongoing movements. Additional information on the balance between inhibitory and excitatory signals within the cortex can be obtained using paired-pulse TMS. This technique uses couples of TMS stimuli—i.e., a conditioning stimulus followed by a test stimulus—with specific combinations of latencies and intensities. For example, a subthreshold conditioning stimulus suppresses the amplitude of MEPs elicited by a suprathreshold test stimulus delivered after 1–5 ms, with respect to MEPs elicited by unconditioned magnetic pulses (i.e., short-interval intracortical inhibition, SICI) [134]. Similarly, a suprathreshold conditioning stimulus lowers MEPs amplitude elicited by a test stimulus delivered with a ~100 ms latency (i.e., long-interval intracortical inhibition, LICI) [135]. These effects are due to the activation of GABAergic interneurons by the conditioning stimulus. Specifically, SICI is caused by the activation of ionotropic GABA_A_ receptors, while LICI depends on metabotropic GABA_B_ receptors [136,137]. All these paired-pulse TMS protocols may facilitate a fine-grained understanding of inhibitory circuits in DCD.

Paired-pulse TMS is also used to investigate interhemispheric connections. A conditioning stimulus delivered to the motor cortex in one hemisphere suppresses corticospinal excitability in the contralateral hemisphere after an interstimulus interval of ~10 ms (short-interval interhemispheric inhibition) or ~40–50 ms (long-interval interhemispheric inhibition) [138,139,140]. Interestingly, interhemispheric inhibition assessed at rest was reduced in a recent study conducted on adult patients with DCD [141]. Further studies assessing interhemispheric inhibition during ongoing movements (and, possibly, in pediatric populations) may help to elucidate the neural correlates of motor coordination difficulties in DCD (e.g., in two hands catching, see [44,45]).

TMS may also help to clarify the neural basis of motor imagery impairments in DCD. In healthy subjects, motor imagery increases corticospinal excitability [142,143,144,145]. This indicates that motor imagery activates the same substrates involved in movement execution. In DCD, the assessment of corticospinal excitability during motor imagery (e.g., in the hand rotation task) may help to investigate the neural basis of the efference copy impairment. In a recent study, TMS pulses were delivered to the motor cortex of adult participants with DCD and neurotypical controls while they were engaged in a hand rotation paradigm [146]. Results showed that, unlike neurotypical controls, participants with DCD did not show any facilitation of corticospinal excitability while engaged in this task. Consistent with previous behavioral findings, these results suggest reduced activation of the neural substrates involved in actual motor control during motor imagery.

Likewise motor imagery, in healthy subjects the observation of both transitive (e.g., grasping of different objects) and intransitive (e.g., arm elevation) actions is associated with an increase in corticospinal excitability that is specific for the muscles involved in the observed action (i.e., motor resonance) [106,147]. This effect indexes the activation of the mirror neuron system in humans. Importantly, motor resonance does not simply consist of a direct-matching mechanism that simulates observed actions in the observer motor repertoire. Rather, MEPs amplitude during action observation reflects complex processing of motor information, including predictions about agent’s intentions [148] and the kinematic similarity between agent’s actions and those encoded in the observer’s motor repertoire [107]. Therefore, this approach may clarify the difficulties in mapping others’ actions that were observed in children with DCD, and possibly help to evaluate the impact of these impairments on motor development.

Finally, more recent applications of TMS are designed to assess the organization of motor synergies. In these studies, the kinematics of TMS-elicited movements are recorded in place of electromyographic responses (i.e., MEPs). This approach captures more global information on the motor output as opposed to MEPs, which only index the activation of individual muscles. By applying reduction of dimensionality techniques (e.g., principal component analysis) it was demonstrated that the dimensionality of movements elicited by TMS pulses delivered over the hand representation of the motor cortex is low [149]. This means that the large number of degrees of freedom available for hand movements is in fact co-controlled by a limited number of motor synergies, organized at cortical level. In light of the studies suggesting motor synergies impairment in DCD, this approach may offer a valuable opportunity for dissecting the neural basis of impaired degrees of freedom control in this condition.

## 7. Conclusions

Previous research conveyed valuable knowledge on motor impairments in DCD. However, little is known about the neural mechanisms of this condition. We propose that TMS of the motor cortex may offer important opportunities to bridge this gap. In turn, this may help to develop more appropriate diagnostic approaches and more effective interventions. TMS-based techniques are safe in children and may allow researchers to dissect cortical circuits at multiple levels, linking their function with behavioral findings.
